# Supplementary material for: Genomic epidemiology of enteropathogenic Escherichia coli in southwestern Nigeria
Source: PLoS Negl Trop Dis. 2025 Aug 22;19(8):e0013442. doi: 10.1371/journal.pntd.0013442 (PMC12404642; doi:10.1371/journal.pntd.0013442)
Supplement: Attachment — Submitted filename: Reviewer comments_EPEC in southwestern Nigeria.docx [file pntd.0013442.s003.docx]

**Genomic epidemiology of enteropathogenic Escherichia coli (EPEC) in southwestern Nigeria**

**Overall comments**

This study investigates the diversity and characteristics of EPEC in Nigeria. Given that diarrhoeal disease caused by EPEC is still a health problem in infants in many places including Africa, it is important to understand the evolution of these pathogens.

The study determined that both classical and non-classical lineages of EPEC are circulating and the predominant pathotype is aEPEC, rather than tEPEC. This follows patterns seen elsewhere, such as in Brazil.

While I feel that the majority of genomic characterisation and analyses in this manuscript are well executed, I have concerns regarding some errors in the manuscript (discrepancies in serotyping, interpretation of E coli pathotypes based on carriage of particular virulence genes, etc).

Additionally, the manuscript structure needs considerable improvement – introduction omits many key points which are either provided in the discussion or not mentioned at all (E coli pathotyping, eae allele typing, etc).

Another concern is the lack of detail in the methods and the absence of a supplementary data file or tables outlining the individual isolate metadata and Biosample numbers.

I would also suggest that the isolate raw reads are uploaded into Enterobase so the isolate sequence types, serotypes, pathotypes, phylogroups, can be confirmed.

Overall, I cannot recommend this manuscript for publication as it is currently written. Multiple major corrections are required, data errors need to be checked/corrected, and additional supplementary data needs to be provided.

**Methods**

Lines 106-112 - How were these E. coli isolates determined to be EPEC ? And therefore included for whole genome analysis ?

Line 109 – missing end square bracket.

Lines 117 – 128 – WGS methods and analyses sections. There are insufficient details provided in these sections.

Was QuBit used for DNA quantification ?

What Illumina platform – MiSeq, NextSeq ? What library prep, etc ?

Was a stand alone SPAdes assembler used ? or a plugin in bioinformatics software ?

No website links or references provided for tools used QUAST, BLAST etc.

Were Resfinder, VirulenceFinder and Plasmidfinder at at CGE (Centre for Genomic Epidemiology) used ? or Resfinder in Galaxy ?

What MLST scheme are you using - Achtman 7 gene or Pasteur scheme ? Please clarify.

What platforms were used for the MLST and serotyping – Galaxy, Github, CGE or Enterobase ? Again no website links for tools.

Reference 24 for MLST seems inappropriate – refers to Neisseria meningitidis – not E. coli. Please correct.

Line 117 – full stop instead of comma i.e. “instructions. The DNA extracts”

Line 130 – correct “(11)O51:H -,” to “(11) O51:H-,”

**Results**

Lines 143-150 – this section is very confusing – how many of the 96 EPEC were from children? And how many of the 96 were from adults ?

Please present isolate numbers as fraction of total (denominator) and as a percentage, e.g., 9/96 (9.4%), etc.

Table 1 – This table is not clear. What are “cases” and what are “controls” ?

Are the age brackets the number of EPEC detected ?

Why does the total number of isolates only add up to 57 ? and not 96 ?

What is the p value significance being measured ?

How were the p-values calculated ?

There should be a statistics section included in the methods.

Lines 153-156 – again, please report numbers as, e.g., 57/96 (59.4%) etc..

As above, further description is required for calculation of p-values, i.e. “.(p<0.05, Table 3).”

Table 2 – again I find this table confusing.

What are “cases” and what are “controls” ? are these EPEC detected ?

Where did the total of 61 come from ?

What do the p-values measure ?

Table 3 – suggest moving the “All EPEC” to the bottom row.

N=805 should be lowercase italicised, i.e. *n* = 805.

Again, add denominators, e.g., 39/805 (4.8%)

What do the p-values measure ?

Line 163 – how was phylogroups determined ? There is no phylotyping details listed in the method ?

Lines 163 – 165 – again, add denominators, e.g., Forty-six of 96 (47.9%) or 20 of 96 (20.8%), etc.

Line 167 – comma after U i.e., “D, E, and U, while”

Figure 1. How was *eae* allele type determined using BLAST – did you have a reference panel of *eae* allele types? The majority of eae alleles in BLASTn are not typed. eae alleles have also been described in several publications by Lacher, Blanco and many others.

Lacher DW, Steinsland H, Whittam TS. Allelic subtyping of the intimin locus (*eae*) of pathogenic *Escherichia coli* by fluorescent RFLP. *FEMS Microbiol Lett*. 2006;261(1):80-7.

Blanco M, Schumacher S, Tasara T, Zweifel C, Blanco JE, Dahbi G, et al. Serotypes, intimin variants and other virulence factors of *eae* positive *Escherichia coli* strains isolated from healthy cattle in Switzerland. Identification of a new intimin variant gene (*eae-*η2). *BMC Microbiol*. 2005;5(1):23.

Please provide additional *eae* typing information (GenBank accessions numbers for reference variants and published refs) in methods/results and/or supplementary table.

Please describe tree construction in methods section.

Line 185 – 16 of 96 (16.7%).

Line 192-193 – 26 of 96 (27.1%), etc..

Line 193 – refers to “Table 6” but there is no Table 6 included in this manuscript. Plus as above, How was *eae* allele type determined ? reference alleles/accession numbers/refs ?

Lines 196-203 ­– This is the first mention of EPEC phylogenetic lineages, i.e. EPEC1, EPEC2, etc. This needs to be addressed in the introduction and reference provided there.

EPEC1 and EPEC2 were first described by Orskov F, Whittam TS, Cravioto A, Orskov I (1990) Clonal relationships among classic enteropathogenic Escherichia coli (EPEC) belong to different O groups. J Infect Dis 162(1):76–81

EPEC1 to EPEC4 were described by Lacher DW, Steinsland H, Blank TE, Donnenberg MS, Whittam TS (2007) Molecular evolution of typical enteropathogenic Escherichia coli: Clonal analysis by multilocus sequence typing and virulence gene allelic proﬁling. J Bacteriol 189(2):342–350. And **Hazen TH, Sahl JW, Fraser CM, Donnenberg MS, Scheutz F et al.** Refining the pathovar paradigm via phylogenomics of the attaching and effacing *Escherichia coli*. *Proc Natl Acad Sci* 2013; 110(31):12810-12815.

Additional EPEC phylogenetic lineages (up to EPEC10) described by Hazen, Tracy H., et al. "Genomic diversity of EPEC associated with clinical presentations of differing severity." *Nature microbiology* 1.2 (2016): 1-9.

The reference cited (Hernandes, 2020) describes additional EPEC lineages BUT only refers to atypical EPEC.

Figure 2 – where did the 67 international strains come from ? How was the tree constructed ? This information should be in the methods and inclusion criteria plus accession numbers for international strains provided.

Line 225 – correct “O101/O162H:33” to “O101/O162:H33”

Lines 214-229 – No mention of other diarrhoeagenic E coli pathotypes in the introduction – need to explain what EHEC, EAEC, EIEC, etc are. Also what virulence genes are associated with particular pathotypes, and those shared by different pathotypes. See Nataro & Kaper 1998, Gomes et al 2016.

A lot of text in this section is not results and needs to be moved to either the introduction or discussion sections.

Figure 3. and text 236-250

The virulence gene “ltcA” is invasion-associated locus A and is associated with multiple DEC including EAEC, EIEC but not usually ETEC. ItcA is not the same as the LT genes (ItA, ItB) found in ETEC. Please correct in figure legend and text.

Characteristic EAEC associated genes include aggR, aap, aggA, agg4A, aar.

The genes air, eilA, capU are found in diverse pathotypes of E coli and not exclusive to EAEC. Please correct in figure legend and text.

Again, A lot of text in this section is not results and needs to be moved to either the introduction or discussion sections.

Line 262 – here (and in abstract) the serotype of the ST7798 isolates is listed as O127:H29, whereas in Figure 1, ST7798 serotype appears to be listed as O80:H2. Note. serotype O127:H29 is not included in the Fig 1 legend. please explain this discrepancy ?

Additionally, all other ST7798 isolates in Enterobase and PubMLST have the serotype Onon-typable:H49, which is different again.

Note – ST7798 isolates in this study and Enterobase are all phylogroup B2 and have kappa eae allele, so this appears correct.

A quick check in Enterobase determined E coli serotypes O127:H29 and O88:H2 are generally phylogroups A or B1.

I would suggest double checking the serotype of your ST7798 isolates – the most reliable option is to upload WGA raw reads into Enterobase, or secondly to check using assembled reads in CGE SeroTypeFinder.

(<https://cge.food.dtu.dk/services/SerotypeFinder/>)

Lines 266-270 – this is discussion – not results.

Fig 4d – no label on vertical axis

**Discussion**

Lines 310-316 – repeats what has already been stated in introduction – combine with intro section.

Line 311 – again, no explanation for defining characteristics of pathotypes (ETEC, EAEC, and EPEC) – is needed in introduction.

Lines 320-322 – reference to Gem study Ingle et al 2016 would be good. Ingle, D. J., Tauschek, M., Edwards, D. J., Hocking, D. M., Pickard, D. J., Azzopardi, K. I., ... & Holt, K. E. (2016). Evolution of atypical enteropathogenic E. coli by repeated acquisition of LEE pathogenicity island variants. *Nature microbiology*, *1*(2), 1-9.

Lines 324-329 – more appropriate for the introduction section, not discussion.

Lines 339-345 – again repeating introduction – please remove and combine with intro section.

Lines 365 – 370 – again should be moved to the introduction section.

**General comments:**

No supplementary data file provided with individual isolate details, metadata, accession IDs, Biosample IDs, etc. There is no way for reviewers to locate individual isolate WGS reads and check the accuracy of the analyses.
