# Supplementary material for: Genomic epidemiology of enteropathogenic Escherichia coli in southwestern Nigeria
Source: PLoS Negl Trop Dis. 2025 Aug 22;19(8):e0013442. doi: 10.1371/journal.pntd.0013442 (PMC12404642; doi:10.1371/journal.pntd.0013442)
Supplement: Attachment — Submitted filename: Reviewer comments_EPEC in southwestern Nigeriav4.docx [file pntd.0013442.s004.docx]

**Genomic epidemiology of enteropathogenic Escherichia coli (EPEC) in southwestern Nigeria**

**Overall comments**

This study investigates the diversity and characteristics of EPEC in Nigeria. Given that diarrhoeal disease caused by EPEC is still a health problem in infants in many places including Africa, it is important to understand the evolution of these pathogens.

The study determined that both classical and non-classical lineages of EPEC are circulating and the predominant pathotype is aEPEC, rather than tEPEC. This follows patterns seen elsewhere, such as in Brazil.

While I feel that the majority of genomic characterisation and analyses in this manuscript are well executed, I have concerns regarding some errors in the manuscript (discrepancies in serotyping, interpretation of E coli pathotypes based on carriage of particular virulence genes, etc).

Additionally, the manuscript structure needs considerable improvement – introduction omits many key points which are either provided in the discussion or not mentioned at all (E coli pathotyping, eae allele typing, etc).

Another concern is the lack of detail in the methods and the absence of a supplementary data file or tables outlining the individual isolate metadata and Biosample numbers.

I would also suggest that the isolate raw reads are uploaded into Enterobase so the isolate sequence types, serotypes, pathotypes, phylogroups, can be confirmed.

Overall, I cannot recommend this manuscript for publication as it is currently written. Multiple major corrections are required, data errors need to be checked/corrected, and additional supplementary data needs to be provided.

**Methods**

Lines 106-112 - How were these E. coli isolates determined to be EPEC? And therefore included for whole genome analysis?

**Author’s response:** The *Escherichia coli* isolates were whole genome sequenced and classified to be EPEC when the virulencefinder outputs identified them to harbour *eae* and/or *bfp* genes. In response to the reviewer’s comment, lines 134-135 have been clearly outlined.

Line 109 – missing end square bracket.

**Author’s response:**

Thanks for your observation the end square bracket has been added. This has been implemented in line 129

Lines 117 – 128 – WGS methods and analyses sections. There are insufficient details provided in these sections.

**Author’s response:** More details have been added to this section in response to the reviewer’s request (lines 147-165)

Was QuBit used for DNA quantification?

**Author’s response:** We find Qubit superior and use this in our current operations but at the time of the study, the DNA were quantified using Nanodrop. This is indicated on line 139 -140.

What Illumina platform – MiSeq, NextSeq ? What library prep, etc ?

**Author’s response**: The isolates were sequenced using the Miseq platform using the 600-cycle v3 reagent kit. The libraries were prepared using the Illumina Nextera XT DNA Library Prep Kit. These are indicated on lines 140-142 of the revised manuscript.

Was a stand-alone SPAdes assembler used ? or a plugin in bioinformatics software ?

**Author’s response:** A stand-alone SPAdes assembler was used on the command line.

No website links or references provided for tools used QUAST, BLAST etc.Were Resfinder, VirulenceFinder and Plasmidfinder at CGE (Centre for Genomic Epidemiology) used ? or Resfinder in Galaxy ?

**Author’s Response:** QUAST was also used on the command line and a reference has been provided on line 160. Resfinder, Virulencefinder, and Plasmidfinder were all used on the commandline and the references have also been provided on line 147-155.

What MLST scheme are you using - Achtman 7 gene or Pasteur scheme ? Please clarify.

What platforms were used for the MLST and serotyping – Galaxy, Github, CGE or Enterobase ? Again no website links for tools.

**Author’s response**: Acthman 7 loci scheme was used for MLST with ARIBA on the commandline and Serotyping was carried out on the commandline too using Ectyper . These are indicated on lines 153-154.

Reference 24 for MLST seems inappropriate – refers to Neisseria meningitidis – not E. coli. Please correct.

**Author’s response:** Thanks for your observation, we have included the corrected MLST reference. This is indicated on line 153

Line 117 – full stop instead of comma i.e. “instructions. The DNA extracts”

**Author’s response:** We have replaced “,” With a “.” to correct the typographical error. This has been implemented in line 138.

Line 130 – correct “(11)O51:H -,” to “(11) O51:H-,”

**Author’s response:** Thank you. The typographical error has been corrected. This has been implemented in line 157.

**Results**

Lines 143-150 – this section is very confusing – how many of the 96 EPEC were from children? And how many of the 96 were from adults?

**Author’s response:** We have reframed the sentences for better clarification. 93 of the EPEC were from children and 3 from adults. This has been implemented in line 178-179.

Please present isolate numbers as fraction of total (denominator) and as a percentage, e.g., 9/96 (9.4%), etc.

**Author’s response:** The isolate numbers and percentages has been presented as suggested. Thanks. This has been implemented in line 185-188.

Table 1 – This table is not clear. What are “cases” and what are “controls” ?

**Author’s response:** The ‘cases’ has been replaced with Individuals with diarrhea while the Controls has been replaced with Controls without diarrhea. This has been implemented in line 189-190

Are the age brackets the number of EPEC detected?

**Author’s response:** The age brackets are the age range of the children that EPEC was isolated from.

Why does the total number of isolates only add up to 57 ? and not 96 ?

**Author’s response:** Thanks for your observations the table shows the age distribution of individuals from whom EPEC were recovered not the number of isolates. This didn’t sum up to 96 because more than one EPEC strain was recovered from some individuals. The second reason for disparities in numbers is that the ages of some participants were not recorded. These have now been added to the table to avoid confusion in line 189

What is the p-value significance being measured?

How were the p-values calculated ?

**Author’s response:** The **p-value** in this table is used to determine if there was a **statistically significant difference** in the distribution of age groups between **cases with EPEC diarrhea** and **controls with EPEC diarrhea.** The **p-value** was calculated using the **Fisher’s Exact Test** (for instances that have an expected value less than 5) or the **Chi-Square Test** (when all expected values were greater than 5). This is now indicated in a footnote on the table (lines 190-191)

There should be a statistics section included in the methods.

**Author’s Response:** Thanks for this observation, we have included this section to the write up. This has been implemented in line 172-175

Lines 153-156 – again, please report numbers as, e.g., 57/96 (59.4%) etc..

As above, further description is required for calculation of p-values, i.e. “.(p<0.05, Table 3).”

**Author’s Response:** We have made the corrections and incorporated them into the table. This has been implemented in line 193-196. However, the statistical description used has been added to the write-up (line 190-191).

Table 2 – again I find this table confusing.

What are “cases” and what are “controls” ? are these EPEC detected ?

Where did the total of 61 come from ?

What do the p-values measure ?

**Author’s response:** Table 2 shows the different studies, the numbers of participants enrolled in each studies and number of EPEC isolated. In response to the reviewer’s request for clarity, we have edited the column headings to read ‘Individuals with diarrhea’ (previously headed ‘cases’) and Controls without diarrhea (previously titled ‘controls’). The p-values were computed using Chi squared or Fisher’s exact test comparing these two groups and an explanatory footnote has been added to clarify this in response to the reviewer’s comment. EPEC were isolated from a total of 61 individuals (there are more isolates because some individuals yielded multiple isolates). The ages of 4 participants were not included in the metadata (Table 1). These have now been added to the table so that there is clarity in the numbers, as requested by the reviewer.

Table 3 – suggest moving the “All EPEC” to the bottom row.

N=805 should be lowercase italicised, i.e. *n* = 805.

Again, add denominators, e.g., 39/805 (4.8%)

What do the p-values measure ?

**Author’s response:** Thanks for your suggestion, We have made the corrections and incorporated them into the Table 3 . The p-value in this table measures whether there is a **statistically significant difference** between **case isolates (diarrhea patients)** and **control isolates (healthy individuals)** for each type of EPEC (Enteropathogenic Escherichia coli).

Line 163 – how was phylogroups determined? There is no phylotyping details listed in the method ?

**Author’s response:** Phylogroups were determined using Waters et al’s CommandLine Tool Ezclermont (1). This has been implemented in line 154-155.

Lines 163 – 165 – again, add denominators, e.g., Forty-six of 96 (47.9%) or 20 of 96 (20.8%), etc.

**Author’s response:** The denominator has been added. This has been implemented in line 209-210.

Line 167 – comma after U i.e., “D, E, and U, while”

**Author’s response:** We have made the correction and this has been implemented in line 213.

Figure 1. How was *eae* allele type determined using BLAST – did you have a reference panel of *eae* allele types? The majority of eae alleles in BLASTn are not typed. eae alleles have also been described in several publications by Lacher, Blanco and many others.

Lacher DW, Steinsland H, Whittam TS. Allelic subtyping of the intimin locus (*eae*) of pathogenic *Escherichia coli* by fluorescent RFLP. *FEMS Microbiol Lett*. 2006;261(1):80-7.

Blanco M, Schumacher S, Tasara T, Zweifel C, Blanco JE, Dahbi G, et al. Serotypes, intimin variants and other virulence factors of *eae* positive *Escherichia coli* strains isolated from healthy cattle in Switzerland. Identification of a new intimin variant gene (*eae-*η2). *BMC Microbiol*. 2005;5(1):23.

Please provide additional *eae* typing information (GenBank accessions numbers for reference variants and published refs) in methods/results and/or supplementary table.

Please describe tree construction in methods section.

**Author’s response:** We sought reference intimin alleles from the literature, retrieved them from Genbank and then used BLAST to query our genomes with them. In the response to the reviewer’s comment, we have provided a more complete explanation and cited the appropriate references. We have also added detailed explanation on how the tree was construted (in lines 161-165)

Line 185 – 16 of 96 (16.7%).

**Author’s response:** We have corrected, and this has been implemented in line 230

Line 192-193 – 26 of 96 (27.1%), etc..

**Author’s response:** We have made the correction and this has been implemented in line 237-238

Line 193 – refers to “Table 6” but there is no Table 6 included in this manuscript. Plus as above, How was *eae* allele type determined ? reference alleles/accession numbers/refs ?

**Author’s response:** Thanks for the observation we have corrected the incorrected Table 6 to Figure 1. The *eae* allele type was determined by blasting the *eae* sequences with the NCBI database and further clicking on the Genbank information to give details on the intimin type (line 151-152). This link provides an example https://www.ncbi.nlm.nih.gov/nucleotide/CP051811.1?report=genbank&log$=nuclalign&blast_rank=1&RID=ZUKCKUHG016&from=120124&to=122928

Lines 196-203 ­– This is the first mention of EPEC phylogenetic lineages, i.e. EPEC1, EPEC2, etc. This needs to be addressed in the introduction and reference provided there.

EPEC1 and EPEC2 were first described by Orskov F, Whittam TS, Cravioto A, Orskov I (1990) Clonal relationships among classic enteropathogenic Escherichia coli (EPEC) belong to different O groups. J Infect Dis 162(1):76–81

EPEC1 to EPEC4 were described by Lacher DW, Steinsland H, Blank TE, Donnenberg MS, Whittam TS (2007) Molecular evolution of typical enteropathogenic Escherichia coli: Clonal analysis by multilocus sequence typing and virulence gene allelic proﬁling. J Bacteriol 189(2):342–350. And **Hazen TH, Sahl JW, Fraser CM, Donnenberg MS, Scheutz F et al.** Refining the pathovar paradigm via phylogenomics of the attaching and effacing *Escherichia coli*. *Proc Natl Acad Sci* 2013; 110(31):12810-12815.

Additional EPEC phylogenetic lineages (up to EPEC10) described by Hazen, Tracy H., et al. "Genomic diversity of EPEC associated with clinical presentations of differing severity." *Nature microbiology* 1.2 (2016): 1-9.The reference cited (Hernandes, 2020) describes additional EPEC lineages BUT only refers to atypical EPEC.

**Author’s response:** Thanks for your observation we have included a section on EPEC lineages on lines 109-111 in the introduction and the requisite references were cited.

Figure 2 – where did the 67 international strains come from ? How was the tree constructed ? This information should be in the methods and inclusion criteria plus accession numbers for international strains provided.

**Author’s response: The 67** international strains were selected from the phylogeny of “Hazen TH, Kaper JB, Nataro JP, Rasko DA. Comparative Genomics Provides Insight into the Diversity of the Attaching and Effacing Escherichia coli Virulence Plasmids. Infect Immun. 2015 Oct;83(10):4103-17. doi: 10.1128/IAI.00769-15. Epub 2015 Aug 3. PMID: 26238712; PMCID: PMC4567640”. The tree was constructed by constructed by mapping reference 67 international EPEC genomes and our genomes with prototypical EAEC 042 strain (FN65554) using an in-house Wellcome Sanger Institute tool for mapping reference to reads - sh16_scripts (https://github.com/sanger-pathogens/sh16_scripts). SNPs were called with snp-sites (Page et al., 2016). Phylogenetic trees were then constructed using IQTree (Minh et al., 2020) using GTR+ASC+G model. This has been implemented in line 161-165 The information of the international strains and accession numbers are attached to the supplementary data 1.

Line 225 – correct “O101/O162H:33” to “O101/O162:H33”

**Author’s response:** We have corrected and this has been implemented in line 270

Lines 214-229 – No mention of other diarrhoeagenic E coli pathotypes in the introduction – need to explain what EHEC, EAEC, EIEC, etc are. Also what virulence genes are associated with particular pathotypes, and those shared by different pathotypes. See Nataro & Kaper 1998, Gomes et al 2016.

A lot of text in this section is not results and needs to be moved to either the introduction or discussion sections.

**Author’s response:** Thanks for your observation, we have included the other diarrhoeagenic E coli pathotypes in the introduction, This has been implemented in line 67-75.

Figure 3. and text 236-250

The virulence gene “ltcA” is invasion-associated locus A and is associated with multiple DEC including EAEC, EIEC but not usually ETEC. ItcA is not the same as the LT genes (ItA, ItB) found in ETEC. Please correct in figure legend and text.

**Author’s response:** The gene encoding the enterotoxigenic Escherichia coli heat-labile enterotoxin A sub-unit is given a mumber of different names in the literature (including ltA, which the reviewer noted). One of these names is ltcA and this is the name used in VirulenceFinder, which we used to identify the gene. In order to avoid confusion, it is best that we used this gene name. FASTA files used as queries by VirulenceFinder can be retrieved from the website and in response to the reviewer’s comment, we have verified that the ltcA alleles are indeed recognizing the heat labile enterotoxin. One is pasted below.

[>ltcA:1:V00275

ATGAAAAATATAACTTTCATTTTTTTTATTTTATTAGCATCGCCATTATATGCAAATGGCGACAGATTATAC

CGTGCTGACTCTAGACCCCCAGATGAAATAAAACGTTTCCGGAGTCTTATGCCCAGAGGTAATGAGTACTTC

GATAGAGGAACTCAAATGAATATTAATCTTTATGATCACGCGAGAGGAACACAAACCGGCTTTGTCAGATAT

GATGACGGATATGTTTCCACTTCTCTTAGTTTGAGAAGTGCTCACTTAGCAGGACAGTATATATTATCAGGA

TATTCACTTACTATATATATCGTTATAGCAAATATGTTTAATGTTAATGATGTAATTAGCGTATACAGCCCT

CACCCATATGAACAGGAGGTTTCTGCGTTAGGTGGAATACCATATTCTCAGATATATGGATGGTATCGTGTT

AATTTTGGTGTGATTGATGAACGATTACATCGTAACAGGGAATATAGAGACCGGTATTACAGAAATCTGAAT

ATAGCTCCGGCAGAGGATGGTTACAGATTAGCAGGTTTCCCACCGGATCACCAAGCTTGGAGAGAAGAACCC

TGGATTCATCATGCACCACAAGGTTGTGGAGATTCATCAAGAACAATCACAGGTGATACTTGTAATGAGGAG

ACCCAGAATCTGAGCACAATATATCTCAGGGAATATCAATCAAAAGTTAAGAGGCAGATATTTTCAGACTAT

CAGTCAGAGGTTGACATATATAACAGAATTCGGGATGAATTATGA]

Characteristic EAEC associated genes include aggR, aap, aggA, agg4A, aar.

The genes air, eilA, capU are found in diverse pathotypes of E coli and not exclusive to EAEC. Please correct in figure legend and text. Again, A lot of text in this section is not results and needs to be moved to either the introduction or discussion sections.

**Author’s Response:** The reviewer is correct to state that eilA and capU, found on EAEC virulence plasmids are not unique to EAEC. But neither are genes on the reviewer’s list such as aap (Monteiro BT, Campos LC, Sircili MP, Franzolin MR, Bevilacqua LF, Nataro JP, Elias WP. The dispersin-encoding gene (aap) is not restricted to enteroaggregative Escherichia coli. Diagn Microbiol Infect Dis. 2009 Sep;65(1):81-4. doi: 10.1016/j.diagmicrobio.2009.05.011. PMID: 19679242). The EAEC pathotype remains to be rigorously defined and therefore we used the gene list by Boisen et al., 2020 (Boisen N, Østerlund MT, Joensen KG, Santiago AE, Mandomando I, Cravioto A, Chattaway MA, Gonyar LA, Overballe-Petersen S, Stine OC, Rasko DA, Scheutz F, Nataro JP. Redefining enteroaggregative Escherichia coli (EAEC): Genomic characterization of epidemiological EAEC strains. PLoS Negl Trop Dis. 2020 Sep 8;14(9):e0008613. doi: 10.1371/journal.pntd.0008613. PMID: 32898134; PMCID: PMC7500659) to define the pathotype, which is the current state of the field.

Line 262 – here (and in abstract) the serotype of the ST7798 isolates is listed as O127:H29, whereas in Figure 1, ST7798 serotype appears to be listed as O80:H2. Note. serotype O127:H29 is not included in the Fig 1 legend. please explain this discrepancy ?

Additionally, all other ST7798 isolates in Enterobase and PubMLST have the serotype Onon-typable:H49, which is different again.

Note – ST7798 isolates in this study and Enterobase are all phylogroup B2 and have kappa eae allele, so this appears correct.

A quick check in Enterobase determined E coli serotypes O127:H29 and O88:H2 are generally phylogroups A or B1.

I would suggest double checking the serotype of your ST7798 isolates – the most reliable option is to upload WGA raw reads into Enterobase, or secondly to check using assembled reads in CGE SeroTypeFinder.

(<https://cge.food.dtu.dk/services/SerotypeFinder/>)

**Author’s response:** Thanks for your observation. ST7798 serotype is O127:H29 as it is in the link (<https://microreact.org/project/xc7LCS3kkpW8Tx8G4yUq2Z-epec-with-location-and-age>) . serotype O127:H29 was mistakenly omitted in Fig 1 legend and this has been added.

Lines 266-270 – this is discussion – not results.

**Author’s response:** Thanks, we have moved the text about ST7798 isolates to the discussion and this has been implemented in line 414-418.

Fig 4d – no label on vertical axis

**Author’s response:** We have included the label on the vertical axis.

**Discussion**

Lines 310-316 – repeats what has already been stated in the introduction – combine with intro section.

**Author’s response:** We have combined the introductory aspect of the discussion on diarrhoeagenic Escherichia coli pathotypes with the introduction section. This has been implemented in lines 67-70. Thanks.

Line 311 – again, no explanation for defining characteristics of pathotypes (ETEC, EAEC, and EPEC) – is needed in the introduction.

**Author’s response:** We have included this in the introduction and this has been implemented in lines 67-76.

Lines 320-322 – reference to Gem study Ingle et al, 2016 would be good. Ingle, D. J., Tauschek, M., Edwards, D. J., Hocking, D. M., Pickard, D. J., Azzopardi, K. I., ... & Holt, K. E. (2016). Evolution of atypical enteropathogenic E. coli by repeated acquisition of LEE pathogenicity island variants. *Nature microbiology*, *1*(2), 1-9.

**Author’s response:** We have included the Ingle et al, 2016 to the reference. This has been implemented in line 345.

Lines 324-329 – more appropriate for the introduction section, not discussion.

**Author’s Response:** Thanks for your suggestions we have moved all this section on EPEC classification, adult volunteer studies, hypervirulent lineages and virulence genes to the introduction. This has been implemented in lines 95-99.

Lines 339-345 – again repeating introduction – please remove and combine with intro section.

**Author’s Response:** Thanks for your suggestions, we have removed it from the discussion.

Lines 365 – 370 – again should be moved to the introduction section.

**Author’s Response:** Thanks for your suggestions we have moved this section to the introduction. This has been implemented in lines 101-104

**General comments:**

No supplementary data file provided with individual isolate details, metadata, accession IDs, Biosample IDs, etc. There is no way for reviewers to locate individual isolate WGS reads and check the accuracy of the analyses.

**Author’s Response:** Thanks for your observation, we have attached 2 supplementary data files containing the individual isolate details, isolates metadata, accession IDs, and Biosample IDs.
