# Supplementary material for: Genomic epidemiology of enteropathogenic Escherichia coli in southwestern Nigeria
Source: PLoS Negl Trop Dis. 2025 Aug 22;19(8):e0013442. doi: 10.1371/journal.pntd.0013442 (PMC12404642; doi:10.1371/journal.pntd.0013442)
Supplement: Attachment — Submitted filename: PLOS EPEC in Nigeria revision comments_June 2025.docx [file pntd.0013442.s005.docx]

**Genomic epidemiology of enteropathogenic Escherichia coli (EPEC) in southwestern Nigeria**

Overall comments for revised manuscript

The revised manuscript is much improved in content and, structure and flows well now. Thank you for providing the additional methods information, references and supplementary data files.

This is an important study in a very under-explored area of diarrhoeal disease in human infants in Nigeria and has global implications.

However, there are still multiple minor errors (generally grammatical) and clarifications required in some sections. Also, please correct the typical EPEC STs listed in the results.

Abstract

Line 8 – need a space between ‘8’ and ‘(8.3%)’

Introduction

Line 67 - add ‘(DEC)’ after ‘Diarrheagenic Escherichia coli’ as you use DEC in line 72.

Line 69 – replace ‘isolates’ with ‘pathotypes’ - i.e., …(EIEC) pathotypes being the most common.

Line 70 – correct grammar error after ‘delineated,, e.g., delineated, which has…

Lines 72-74 I would change the following:

“Other DEC pathotypes, enterohemorrhagic E. coli (EHEC), Shiga toxin-producing E. coli (STEC) and diffusely adherent E. coli (DAEC), are, like EPEC, ETEC, EIEC, STEC and EHEC, differentiated based on…”

Change to:

“Other DEC pathotypes, including, enterohemorrhagic E. coli (EHEC), Shiga toxin-producing E. coli (STEC) and diffusely adherent E. coli (DAEC), are also differentiated based on…”

Having all the extra acronyms makes it too confusing.

Lines 75-77 “EPEC has more or less been eliminated as a public health threat in Europe and North America (9) but continues to cause infections in Asian, South American and African settings (10–12).”

This repeats what is in Lines 69 to 71.

I suggest removing Lines 69-71 and combining with Lines 75-77, as this eliminates duplication and flows better with the following text.

Lines 79 – 82 “focus on only these so-called classical, serovarieties” . Please provide reference(s) for “classical” EPEC serotypes.

Line 90 – please add ‘human’ and ‘HEp-2 cells’, i.e. cultured human epithelial cells (HEp-2)

Line 91 – This reference (20) does not appear to be correct – please use Vial, P., Mathewson, J., DuPont, H., Guers, L., Levine, M., 1990. Comparison of two assay methods for patterns of adherence to HEp-2 cells of *Escherichia coli* from patients with diarrhea. J. Clin. Microbiol. 28, 882–885. (Or similarly appropriate reference).

Line 92 – please lesions and change is to are. i.e., and-effacing lesions are produced…

Line 92, please add ‘including intimin’, i.e., collection of virulence factors, including intimin, located…

Line 101 – Intimin is not the gene, please correct to “Intimin, encoding by…”

Lines 101 to 104 – suggest shifting this to Line 93 – i.e. immediately after ‘Effacement (LEE) pathogenicity island (21).’

i.e., “…Effacement (LEE) pathogenicity island (21). Intimin, encoded by the eae gene that is part of the LEE, is required for intestinal colonization in the host and the EPEC defining attaching-and-effacement phenotype. Different intimin subtypes are classified based on the variable 280-amino acid C-terminal sequence of intimin (Int280) (27,28).”

Line 108 – combine into 1 sentence, i.e., “…identification, we…”

Line 108 – clarify ‘parts’ – other parts of the world ? other countries ?

Lines 115 -117 “We report a predominance of atypical EPEC isolates, belonging to non-classical serotypes, and retrospective identification of two outbreaks, one of which involved at least 19 children.”

This is results – please rephrase as what was undertaken in the study, not the results of the study.

Methods

Lines 128 -130 – I am confused by the number of studies are “fourth” studies are referred to twice “Three of these studies were conducted in Ibadan, Oyo State [one unpublished and (13,14)] and the **fourth** in Ile-Ife in adjoining Osun State (33). A **fourth study**, still in analysis, …”

Please clarify.

Line 141 – please add SPAdes reference.

Line 149 – Capital F in Resfinder, i.e., ResFinder

Line 157 – add “as” i.e., others, as non-classical

Line 160 -add phylogenetic, i.e., An EPEC phylogenetic tree…

Line 160 – ‘refernce’ is in an incorrect position in the sentence. Please change to, “mapping 67 international **reference** EPEC genomes”

Results

Line 178 – I’m not sure what the authors mean by “line listed” – do they mean individual isolates ? please clarify.

Lines 182-184 – I would remove the ‘-‘ symbol before the numbers, as it could be interpreted as a minus symbol (i.e., a negative number).

Line 187 – I presume the authors mean statistically significantly associated with diarrhea ? Please clarify in text.

Line 189 – Please add full stop at end of sentence.

Lines 196-197 “However, typical EPEC, which were uncommon overall, were recovered from 9/96 (1.1%) individuals with diarrhea and 4/96 (0.3%) apparently healthy controls (p<0.05, Table 3).” This is an important result – please add additional text to results to state that carriage of tEPEC was significantly associated with clinical disease i.e. diarrhea.

Table 2 – please add (Reference) to Source studies column , i.e. “Source studies (Reference)”

Line 223 – remove space between ST and 517

Figure 1

ST7798 isolates are shown as atypical EPEC – shouldn’t they be typical EPEC (as stated in the text - Lines?

Lines 225-226 – Incorrect STs listed as typical EPEC:

“Typical EPEC isolates belonged to ST28, ST206 (and related ST4128) or ST7798” should also have ST2346 and ST328 listed as typical EPEC. ST7798 should be removed as they are all aEPEC (according to the supp data file and figure 1).

Line 227 – add with before all, i.e., and ST2346 (O142:H34), with all ST28 EPEC

Line 229 – should be “both *bfp* and *eae* genes”

Lines 234-235 “non-classical O151/O118:H5 (n=4) were all typical EPEC while other serotypes were atypical EPEC.” I find this confusing – please correct grammar to clarify.

Lines 243-245 – “MLST and then whole genome sequence approaches have since confirmed these EPEC lineages and uncovered a total of 15 EPEC phylogenomic groups (12,30–32,47).”

This should be in the introduction – not in the results. Please remove and incorporate into the appropriate introduction section (Lines 109-111).

Lines 245 -246 “We included representatives of each of these groups in the phylogeny displayed in Fig 2 and their properties are listed in Table S2.” – this is methods and should be moved to the methods section and/or figure 2 legend.

Lines 248-249 – as previously mentioned – remove “-“ as it looks like a minus (negative number) symbol.

Figure 2 – bottom of outer ring is missing. I also can’t read the isolate IDs – please ensure image is of sufficient resolution so the reader can identify isolate names. Also, please label the ring categories on the image as well as in the figure legend.

Also remove the ‘?’ symbol after lineage in the figure.

Is it possible to highlight the isolates from this study somehow ? This would make it easier to see where they fit in with other lineages.

Lines 255-256 – The description of rings here does not match with the Figure 2 legend –

I.e. “the isolation sources, the middle circle represents the strain types, with typical in green, outer circle represent the phylogenomic lineages”.

What is the isolation source – is this location ?

What is strain type – is this different to isolate type in the figure legend ?

Please clarify in ring descriptions in figure legend and in the figure.

Lines 268-275 – Please just report the results for your ST378 isolate in this results section. And move the text regarding the relationship with the Brazilian clone to the discussion.

Lines 287-289 – again please remove the “-“ symbols.

Lines 294-296 “The Afa/Dr fimbriae define diffusively adherent E. coli (DAEC) (but are also found in other pathotypes) and are associated with IL-8 secretion in diarrhea, a marker of inflammation.” This is not results, please move to introduction or remove altogether.

Add comment re afa significance only, eg., Five isolates carried afa genes (defining genes for DAEC). – Note. The acronym DAEC is already defined in the introduction.

Lines 300-301 – rephrase sentence, eg. Change to “Altogether, 18 (18.8%) isolates in the study carried genes associated with other diarrheagenic E. coli pathotypes, in addition to the EPEC virulence genes.”

Line 316 & 317 – remove space between ST and 517.

Line 316 n = 25 should be *n* = 25 (italics)

Line 319 – should be eae epsilon gene

Discussion

Line 356 – add “and” after Africa

Lines 373-374 – I find this sentence confusing – please re-write to clarify the point.

Lines 374-375 “Specific O:H types are more reliable markers of EPEC” – Do the authors mean historically reliable markers ? Please clarify.

Line 381 – Please The at the start and a space after 8., i.e., The majority, 88 (91.7%)

Line 397 – add space after 38

Line 401- does “prime” mean “target” in this context – please clarify.

Line 430 – remove / between H19 and ST571

Line 434 – replace fullstop with comma, i.e., this study, however, …
