# Supplementary material for: Genomic epidemiology of enteropathogenic Escherichia coli in southwestern Nigeria
Source: PLoS Negl Trop Dis. 2025 Aug 22;19(8):e0013442. doi: 10.1371/journal.pntd.0013442 (PMC12404642; doi:10.1371/journal.pntd.0013442)
Supplement: Attachment — Submitted filename: Response to reviewers comments V6.docx [file pntd.0013442.s006.docx]

Reviewer #1: I do not understand the reason the authors did not respond to my prior comments but without a point-by-point responses to my queries, I cannot evaluate the manuscript. here are the prior comments for their reference.

*We apologise for not fully addressing Reviewer #1’s concerns and for the difficulty in following our response to individual reviewer comments. We appreciate you bringing this to our attention and are grateful for the opportunity to now address each of the comments point by point. Please find our detailed responses below. In many instances, we have gone on to further address this reviewer’s comments following our last version.*


Major points:

1. The reviewer requested that it might be important to clarify the differences between classical and non-classical EPEC vs typical and atypical EPEC (e.g. table 3). For the not expert reader, this is confusing. Do classical EPEC include tEPEC and aEPEC? How about non-classical?

*The terms classical and non-classical are defined in the introduction on lines 77-87 and typical vs atypical EPEC in lines 95-102. Both sections were expanded in response to the reviewer’s comment at the last round. In response to the reviewer’s comment about this being confusing for non-experts, we have additionally added a column to Table 3 that summarizes the criteria for each category.*

2. The reviewer asked that at the Introduction and discussion, it might be important to discuss the trend happening worldwide of more aEPEC causing more outbreaks and cases than tEPEC. Is Africa seeing the same trend as in Europe? Such comparisons have been made in Clin Microb Infect, 2015. 21:729-734.

*On lines 102-104 and 368-370 we have included a short overview of atypical EPEC outbreaks. In response to the reviewer’s comment, we have highlighted the recent upswing in these detections but with the caveat that at least some of this may be connected to better capacity to detect aEPEC and non-classical strains.*

3. Lanes 135-138, results (lanes 304-307) and Fig 4D, unfortunately the sample size is so small that conclusions associated with stunting and wasting are not fully supported. It is well known from the GEM study that diarrheal infectious by more than one pathogen, not just EPEC, are associated with stunting and wasting and in the current study they cannot rule out the presence of a parasitic or viral infection or other bacteria in these patients. Please revise.

*We agree with the reviewer that other pathogens and/or other factors were involved in diarrhoea seen in children infected with ST517 because more children infected with the outbreak strain were asymptomatic. We are not suggesting that EPEC caused the wasting, simply observing that children with nutritional deficiencies were more likely to be symptomatic in this outbreak. This could be a cause, an effect, or neither. As the reviewer states, the sample size is to small to make conclusions from the observations. We have not done so: simply pointed out the observations in the hope that they can prompt future research. It is especially important to do so in a study done in Nigeria where deaths from diarrhoea among malnourished children are among the highest in the world (Grellety et al. 2025). As the reviewer’s comment suggests that presenting the data could lead to it being misread as a significant effect, we have clearly stated that it is not on lines 451-453.*

5. The reviewer said in Fig 1 and lanes 244-246, is the presence of one gene (ltcA) sufficient to define the strain as EPEC-ETEC hybrid strain?

*Yes, the presence of a single gene from one pathotype is sufficient to describe the strain as hybrid if it belongs to another pathotype but only if that gene is definitive for the pathotype. Strains that carry genes that define multiple pathotypes are classified as hybrid if they have defining genes for two or more pathotypes and these strains evolve when virulence genes on plasmids or phage are acquired by a strain belonging to an existing pathotype (Bai et al., 2019, Rasko et al, 2011). Since* ltcA *and* eae/bfp *define their respective pathotypes, strains that have both are hybrids. Theoretically, any virulence factor on a mobile element can move around and thus the definition of hybrids privileges better known virulence loci that are the basis for defining pathotypes. There are strains that harbor genes associated with another pathotype (e.g.* astA *can be found in many pathotypes but was discovered in EAEC first), but these do not fit the definition of hybrid, in terms of how the term is used. In response to the reviewer’s question, we have added the requirement that the locus define the pathotype to the legend of Fig. 3.*

6. The reviewer observed that in Lanes 202-203 and Fig 2, the closely relatedness to Gambian samples is not evident from the data as presented.

*Thanks for your observation we have included a X in red colour to show this on fig 2, and indicated this in the legend.*

Minor points:
1. The reviewer said in the Abstract, lane 38, “…were the most prevalent…” should be corrected.

*On line 38, we have input the correction (line 38)*

2. The reviewer in Lane 68, asked if we meant “pathogroups” instead of “subcategories.

*‘Subcategories’ has been replaced with ‘pathotypes’ in our earlier revision of the manuscript (line 73)*
3. The reviewer asked what “The serovarieties represent in Line 75.

*Serovarieties represent clades descended from a common ancestor that produce similar O (somatic) and H (flagella) antigens. This was addressed at last review (lines 80-82).*

4. The reviewer observed that in Lane 188 and fig 1, the serotype O142:H34 is missing from Fig. 1 list of serotypes.

*Thanks for your observation we have added this into the Fig 1 legend.*

5. The reviewer in Lane 208 ask us to write it as “isolation sources and location…”

*We have inputted this correction in line 257.*

6. The reviewer said in Fig. 2 the external ring, at the bottom of Fig. 2 is not fully visible.

*Thanks for your observation we have corrected that with another image.*

7. The reviewer observed that the color code in fig 4B is confusing because it uses green and yellow in most conditions tested. Perhaps the study type and age range could be depicted with a different color.

*We have changed the colour code for study type and age range.*

8.The reviewer asked us to replace Lanes 310-311 with, “…with isolates of ETEC, EAEC and EPEC being…”.

*That aspect of the write-up has been moved and reframed into the introduction.*

9. The reviewer asked in Lane 315, what do you mean with “insensitive”? do you mean “absent”?.

*In response to the reviewer’s comment, we have changed ‘insensitive’ to ‘low sensitivity’ on line 356 to 357 of the revision*

10.The reviewer asked Lane 320 to be replaced with, “…of the EPEC pathogroup…”.

*On line 361-362 we have corrected it.*
11. Lane 340, “…12 EPEC serogroups…”

*This aspect has been removed in response to a comment another reviewer in the last revised manuscript.*

12. The reviewer commented that in Lane 379 “unfamiliar to science”?

*We implied that these lineages have always existed but been unknown to the scientific community. In response to the reviewer’s comment, We have revised the wording in the manuscript to read ‘under-characterized in the literature’(lines 419).*

13. The reviewer requested that Lane 382, should be replaced with “…EPEC, and more generally, to the Ecoli…”.

*We have restructured the sentence in line with the reviewer’s suggestion on line 423.*

Overall comments for revised manuscript

The revised manuscript is much improved in content and, structure and flows well now. Thank you for providing the additional methods information, references and supplementary data files.

This is an important study in a very under-explored area of diarrhoeal disease in human infants in Nigeria and has global implications.

*We thank the reviewer for these positive comments*

Abstract

1. The reviewer asked us to put a space between ‘8’ and ‘(8.3%), On line 38

*The correction has been made.*

Introduction

2. The reviewer requested that in line 67, we should add ‘(DEC)’ after ‘Diarrheagenic Escherichia coli’ as you use DEC in line 72.

*Thanks for your observation we have added it in line 67.*

3. The reviewer asked us to replace ‘isolates’ with ‘pathotypes’ - i.e., …(EIEC) pathotypes being the most common in line 69.

*We have corrected that in lines 69*

4. The reviewer in line 70 requested that we correct the grammatical error after ‘delineated,, e.g., delineated.

*The sentence has been corrected and reframed in line 77.*

5. The reviewer suggested that in Lines 72-74, S/he would change the following: “Other DEC pathotypes, enterohemorrhagic E. coli (EHEC), Shiga toxin-producing E. coli (STEC) and diffusely adherent E. coli (DAEC), are, like EPEC, ETEC, EIEC, STEC and EHEC, differentiated based on…” to “Other DEC pathotypes, including, enterohemorrhagic E. coli (EHEC), Shiga toxin-producing E. coli (STEC) and diffusely adherent E. coli (DAEC), are also differentiated based on…”.

*Thanks for your observation, we have corrected these as suggested, as in lines 73-75.*

6. The reviewer suggested that in Lines 75-77 “EPEC has more or less been eliminated as a public health threat in Europe and North America (9) but continues to cause infections in Asian, South American and African settings (10–12).” This repeats what is in Lines 69 to 71. I suggest removing Lines 69-71 and combining with Lines 75-77, as this eliminates duplication and flows better with the following text.

*Thanks for your suggestion we have implemented this in Lines 69-72.*

7. The reviewer requested that we should provide reference(s) for “classical” EPEC serotypes on Lines 79 – 82 “focus on only these so-called classical, serovarieties” .

*We have supplied the references there on line 83.*

8. The reviewer requested that we please add ‘human’ and ‘HEp-2 cells’, i.e. cultured human epithelial cells (HEp-2) in Line 90.

*We have replaced epithelial cells with human epithelial cells (HEp-2) on line 88.*

9. The reviewer suggested that in Line 91 – This reference (20) does not appear to be correct – please use Vial, P., Mathewson, J., DuPont, H., Guers, L., Levine, M., 1990. Comparison of two assay methods for patterns of adherence to HEp-2 cells of *Escherichia coli* from patients with diarrhea. J. Clin. Microbiol. 28, 882–885. (Or similarly appropriate reference).

*This is indeed the wrong reference and we apologize for the error. We have cited the reference recommended by the reviewer in line 90.*

10. The reviewer requested that in Line 92 – lesions and change is to are. i.e., and-effacing lesions are produced.

*We have replaced this appropriately in the sentence in line 90.*

11. The reviewer also requested that we please add ‘including intimin’, i.e., ‘collection of virulence factors, including intimin, located’’.

*We have included this in the sentence in line 91.*

12. The reviewer stated that in Line 101 – Intimin is not the gene, please correct to “Intimin, encoding by”.

*We have corrected it in line 91 and throughout the manuscript.*

13. The reviewer suggested that in Lines 101 to 104 – shifting Line 93 – i.e. immediately after ‘Effacement (LEE) pathogenicity island (21).’ i.e., “…Effacement (LEE) pathogenicity island (21). Intimin, encoded by the eae gene that is part of the LEE, is required for intestinal colonization in the host and the EPEC defining attaching-and-effacing phenotype. Different intimin subtypes are classified based on the variable 280-amino acid C-terminal sequence of intimin (Int280) (27,28).”

*Thanks for your suggestion, we have rearranged the sentence and it is now in line 94-95.*

14. The reviewer requested that in Line 108 we should combine into 1 sentence, i.e., “…identification, we…”.

*We have combined the sentence into one on line 109.*

15. The reviewer in Line 108 required clarification if it is ‘parts’ – other parts of the world or other countries?

*We have clarified the sentence for better understanding in line 109.*

16. On Lines 115 -117, the reviewer observed that “We report a predominance of atypical EPEC isolates, belonging to non-classical serotypes, and retrospective identification of two outbreaks, one of which involved at least 19 children.” These is results – please rephrase as what was undertaken in the study, not the results of the study.

*Thanks for your observation, we have reframed the sentence in lines 117-119.*

Methods

17. The reviewer was confused in Lines 128 -130 – on the number of studies are “fourth” studies are referred to twice “Three of these studies were conducted in Ibadan, Oyo State [one unpublished and (13,14)] and the fourth in Ile-Ife in adjoining Osun State (33). A fourth study, still in analysis, …” Please clarify.

*On lines 129-133 the sentence has been rephrased for clearer understanding.*

18. The reviewer requested that we please add a SPAdes reference on Line 141.

*We have added the reference as requested on line 144.*

19. The reviewer on Line 149 asked that Resfinder, should be replaced with ResFinder.

*Thanks for your observation we corrected it in line 152.*

20. The reviewer requested that we add “as” i.e., others, as non-classical in line 157.

*We have added the as to the sentence in line 160.*

21. In Line 160 the reviewer asked that we add phylogenetic, i.e., An EPEC phylogenetic tree to the sentence.

*We have incorporated this correction into line 163.*

22. Line 160 the reviewer asked to please change to, “mapping 67 international reference EPEC genomes”.

*We have incorporated this correction into line 163.*

Results

23. The reviewer in Line 178 – do not understand what the authors mean by “line listed” – do they mean individual isolates? please clarify.

*The phrase “line-listed” is an epidemiological one that implies individual records and in line 182 was used to refer to individual isolates. However, in response to the reviewer’s comment, it has been deleted for better clarification.*

24. The reviewer in Lines 182-184 suggested the removal of ‘-‘ symbol before the numbers, as it could be interpreted as a minus symbol (i.e., a negative number).

*Thanks for your suggestion we have removed all the ‘-‘ symbol now in line 186-188.*

25. The reviewer in Line 187 presumed that the authors meant statistically significantly associated with diarrhea ? Please clarify in text.

*We meant not statistically significantly as the p-value obtained were greater than 0.05 as stated in line 191.*

26. The reviewer requested us to add full stop at the end of the sentence in Line 189.

*Thanks for your observation we have added the full stop to the sentence (Line 193).*

27. The reviewer stated that in Lines 196-197 “However, typical EPEC, which were uncommon overall, were recovered from 9/96 (1.1%) individuals with diarrhea and 4/96 (0.3%) apparently healthy controls (p<0.05, Table 3).”

*This is an important result – please add additional text to results to* *state that carriage of tEPEC was significantly associated with clinical disease i.e. diarrhea. On line 201-202, we have inserted the sentence to highlight this important point.*

28. The reviewer requested in Table 2 that, add (Reference) to Source studies column , i.e. “Source studies (Reference)”.

*On line 204 we have added the word “reference” to the source studies.*

29. In Line 223 the reviewer requested that we remove space between ST and 517.

*We have removed the space between ST and 517 as stated in line 229.*

Figure 1

30. The reviewer asked that the ST7798 isolates are shown as atypical EPEC – shouldn’t they be typical EPEC (as stated in the text - Lines?).

*ST7798 are atypical EPEC and they were represented as atypical in the figure, and this has been corrected and removed in the text on line 232. Thanks for your observation.*

31. Lines 225-226 – Incorrect STs listed as typical EPEC: “Typical EPEC isolates belonged to ST28, ST206 (and related ST4128) or ST7798” should also have ST2346 and ST328 listed as typical EPEC. ST7798 should be removed as they are all aEPEC (as indicated in the supp data file and figure 1).

*Thanks for your observation, we apologize for the error, we have removed ST7798 (line 232) as that was a typological error from us and ST2346 and ST328 has been listed as typical EPEC on line 232*

32. The reviewer in Line 227 requested that we add with before all, i.e., and ST2346 (O142:H34), with all ST28 EPEC.

*We have added this in line 234.*

33. The reviewer stated that Line 229 should read “both *bfp* and *eae* genes”.

*We have corrected this in line 236.*

34. The reviewer in Lines 234-235 is confused if “non-classical O151/O118:H5 (n=4) were all typical EPEC while other serotypes were atypical EPEC.”

*Thanks for your observation. The typical EPEC isolates belonged to both classical serotypes O119:H6 (n=5), O142:H34 (n=3), and non-classical O151/O118:H5 (n=4), while other serotypes were atypical EPEC. We have reframed the sentence for better understanding (lines 240-242).*

35. The reviewer requested that in Lines 243-245 – “MLST and then whole genome sequence approaches have since confirmed these EPEC lineages and uncovered a total of 15 EPEC phylogenomic groups (12,30–32,47).” This should be in the introduction – not in the results. Please remove and incorporate into the appropriate introduction section.

*We have moved this sentence to the introduction section lines 111-113.*

36. The reviewer in Lines 245 -246 requested that the sentence “We included representatives of each of these groups in the phylogeny displayed in Fig 2 and their properties are listed in Table S2.” – this is methods and should be moved to the methods section and/or figure 2 legend.

*This sentence has been moved and incorporated int the methodology section of the manuscript (Line 163-164)*

37. The reviewer suggested that Lines as previously mentioned – remove “-“ as it looks like a minus (negative number) symbol.

*We have removed the “-“ from the sentence (lines 252-253)*

38. The reviewer in Figure 2 observed that the bottom of outer ring is missing and also can’t read the isolate IDs – please ensure image is of sufficient resolution so the reader can identify isolate names. Also, please label the ring categories on the image as well as in the figure legend. Also remove the ‘?’ symbol after lineage in the figure. Is it possible to highlight the isolates from this study somehow? This would make it easier to see where they fit in with other lineages.

*Thanks for your observation, we have created another image for better visualization, this include some of isolates IDs and also labelled the ring on the figure legend.*

39. The reviewer in Lines 255-256 said that the description of rings here does not match with the Figure 2 legend – I.e. “the isolation sources, the middle circle represents the strain types, with typical in green, outer circle represent the phylogenomic lineages”. What is the isolation source – is this location? What is strain type – is this different to isolate type in the figure legend? Please clarify in ring descriptions in figure legend and in the figure.

*We have reframed the legend to match the descriptions as suggested, the isolate source means the location and it is written now as the isolation sources and location, the strain type also means the isolate type and this has been replaces with the strain types in the figure legend (line 257-263)*

40. The reviewer in Lines 268-275 requested that “Please just report the results for your ST378 isolate in this results section and move the text regarding the relationship with the Brazilian clone to the discussion”.

*We have moved this to the discussion section of the manuscript (lines 404-406).*

41. The reviewer in Lines 287-289 asked that the “-“ symbols should be removed.

*We have removed all the “-“ symbols (Lines 290-291)*

42. The reviewer in stated that “Lines 294-296 the Afa/Dr fimbriae define diffusively adherent E. coli (DAEC) (but are also found in other pathotypes) and are associated with IL-8 secretion in diarrhea, a marker of inflammation. This is not results, please move to introduction or remove altogether. Add comment that are significance only, eg., Five isolates carried afa genes (defining genes for DAEC). – Note. The acronym DAEC is already defined in the introduction”.

*We have removed this sentence completely from the result section and from the manuscript.*

43. The reviewer stated that in Lines 300-301 we should rephrase sentence, eg. Change to “Altogether, 18 (18.8%) isolates in the study carried genes associated with other diarrheagenic E. coli pathotypes, in addition to the EPEC virulence genes.”

*On line 301-302, we have input the rephrased sentence into the write-up.*

44. The reviewer asked that in Line 316 & 317 – remove space between ST and 517 , Line 316 n = 25 should be *n* = 25 (italics).

*Thanks for your observation, we have corrected this in lines 317-318.*

45. The reviewer requested that we change the intimin to eae epsilon gene (Line 319).

*We have corrected this in line 320.*

Discussion

46. The reviewer in Line 356 requested that we add “and” after Africa.

*We have added this to the sentence (line 357)*

47. The reviewer found the sentence in Lines 373-374 confusing – please re-write to clarify the point.

*We have reframed the sentence and made it clearer in lines 376-379.*

48. The reviewer requested for clarification of “Specific O:H types are more reliable markers of EPEC” in Lines 374-375 – Do the authors mean historically reliable markers ? Please clarify.

*Thank you for the comment. Yes, we meant that specific O:H types have historically been used as reliable markers than O types alone for identifying EPEC,* *particularly classical strains. While molecular tools are now more commonly used for classification, certain O:H serotypes remain closely associated with EPEC and have been useful in distinguishing them from other diarrhoeagenic E. coli pathotypes. We have clarified this in the revised manuscript. (lines 377-381)*

49. The reviewer asked that we put The at the start and a space after 8. in Line 381, i.e., The majority, 88 (91.7%).

*In line 387, we have done the correction.*

50. The reviewer requested that we add space after 38 in Line 397.

*We have added the space after the number (line 403)*

51. The reviewer asked us to clarify if “prime” means “target” in Line 401.

*The word prime means target for amplification. As this is the reviewer’s preference, we have edited line 409 accordingly*

52. The reviewer in Line 430 requested us to remove / between H19 and ST571.

*We have removed the / between the H19 and ST57 in line 439. Thanks*

53. The reviewer in Line 434 asked us to replace full stop with comma, i.e., this study, however.

*We have made this correction in line 443.*

**Literature cited in the response**

- Grellety E, Simons E, Mousset M, et al. Diagnostic criteria for severe acute malnutrition and fatal outcomes in children aged 6-59 months, Nigeria. Bull World Health Organ. 2025 Jul 1;103(7):418-428. doi: 10.2471/BLT.24.292143.
- Bai, X., Zhang, J., Ambikan, A. *et al.* Molecular Characterization and Comparative Genomics of Clinical Hybrid Shiga Toxin-Producing and Enterotoxigenic *Escherichia coli* (STEC/ETEC) Strains in Sweden. *Sci Rep***9**, 5619 (2019). <https://doi.org/10.1038/s41598-019-42122-z>
- Rasko, D. A. *et al*. Origins of the *E. coli* strain causing an outbreak of hemolytic-uremic syndrome in Germany. *N Engl J Med* **365**, 709–717, <https://doi.org/10.1056/NEJMoa1106920> (2011).
